# Cerebrospinal fluid, plasma, and saliva in the BioFIND study: Relationships among biomarkers and Parkinson's disease Features

**DOI:** 10.1002/mds.27232

**Published:** 2017-12-04

**Authors:** Jennifer G. Goldman, Howard Andrews, Amy Amara, Anna Naito, Roy N. Alcalay, Leslie M. Shaw, Peggy Taylor, Tao Xie, Paul Tuite, Claire Henchcliffe, Penelope Hogarth, Samuel Frank, Marie‐Helene Saint‐Hilaire, Mark Frasier, Vanessa Arnedo, Alyssa N. Reimer, Margaret Sutherland, Christine Swanson‐Fischer, Katrina Gwinn, The Fox Investigation of New Biomarker Discovery, Un Jung Kang

**Affiliations:** ^1^ Section of Parkinson Disease and Movement Disorders, Department of Neurological Sciences Rush University Medical Center Chicago Illinois USA; ^2^ Division of Movement Disorders, Department of Neurology Columbia University Medical Center New York New York USA; ^3^ Department of Neurology University of Alabama at Birmingham Birmingham Alabama USA; ^4^ The Michael J. Fox Foundation for Parkinson's Research New York New York USA; ^5^ Department of Pathology & Laboratory Medicine, Institute on Aging, Center for Neurodegenerative Disease Research University of Pennsylvania School of Medicine Philadelphia PA USA; ^6^ BioLegend Inc Dedham USA; ^7^ Parkinson Disease and Movement Disorder Program, Department of Neurology University of Chicago Chicago Illinois USA; ^8^ Department of Neurology University of Minnesota Minneapolis Minnesota USA; ^9^ Department of Neurology Weill Cornell Medical College New York New York USA; ^10^ Department of Molecular and Medical Genetics Oregon Health & Science University Portland Oregon USA; ^11^ Department of Neurology Boston University School of Medicine Boston Massachusetts USA; ^12^ National Institute of Neurological Disorders and Stroke National Institutes of Health Bethesda Maryland USA

**Keywords:** alpha‐synuclein, amyloid, cerebrospinal fluid, postural instability gait difficulty, tau

## Abstract

**Objective:** Examine relationships among neurodegenerative biomarkers and PD motor and nonmotor symptoms.

**Background:** CSF alpha‐synuclein is decreased in PD versus healthy controls, but whether plasma and saliva alpha‐synuclein differentiate these groups is controversial. Correlations of alpha‐synuclein among biofluids (CSF, plasma, saliva) or biomarkers (eg, beta‐amyloid, tau [total, phosphorylated]) are not fully understood. The relationships of these biomarkers with PD clinical features remain unclear.

**Methods:** BioFIND, a cross‐sectional, observational study, examines clinical and biomarker characteristics in moderate‐advanced PD and matched healthy controls. We compared alpha‐synuclein concentrations across diagnosis, biofluids, and CSF biomarkers. Correlations of CSF biomarkers and MDS‐UPDRS, motor phenotype, MoCA, and rapid eye movement sleep behavior disorder questionnaire scores in PD were examined.

**Results:** CSF alpha‐synuclein was lower in PD versus controls (*P* = .01), controlling for age, gender, and education. Plasma and saliva alpha‐synuclein did not differ between PD and controls, and alpha‐synuclein did not significantly correlate among biofluids. CSF beta‐amyloid_1‐42_ was lower in PD versus controls (*P* < .01), and correlated weakly with MoCA recall scores (*r* = 0.23, *P* = .02). CSF alpha‐synuclein was lower in the postural instability/gait difficulty phenotype than other motor phenotypes (*P* < .01). No CSF biomarkers predicted or correlated with total motor or rapid eye movement sleep behavior disorder scores. CSF alpha‐synuclein correlated with beta‐amyloid_1‐42_, total‐tau, and phosphorylated‐tau (*r* = 0.41, 0.81, 0.43, respectively; *P*s < .001).

**Conclusion:** Lower CSF alpha‐synuclein is associated with diagnosis and motor phenotype in moderate‐advanced PD. Plasma and saliva alpha‐synuclein neither correlate with CSF alpha‐synuclein, nor distinguish PD from controls. CSF beta‐amyloid_1‐42_ remains a potential biomarker for cognitive impairment in PD. © 2017 The Authors. Movement Disorders published by Wiley Periodicals, Inc. on behalf of International Parkinson and Movement Disorder Society.

Biomarkers have the potential to play a role in establishing PD diagnosis, understanding disease progression or disease‐related features, and monitoring therapeutic effects. In PD, candidate biofluids include cerebrospinal fluid (CSF), plasma/serum, saliva, and urine, among others.^1^, ^2^, ^3^ Alpha‐synuclein (α‐syn) holds promise as a biomarker because it is a major component of Lewy bodies, can be found in peripheral tissues and body fluids, is readily secreted into extracellular spaces, and in part can be detected in exosomes.^4^ Studies consistently demonstrate reduced CSF total α‐syn levels in PD when compared with healthy controls (HC).^1^, ^5^, ^6^ Because of the invasive nature of acquiring CSF, more readily accessible biofluids, such as blood or saliva, are attractive alternatives. Red blood cells express high levels of α‐syn, but reports of plasma/serum α‐syn in PD when compared with HC have yielded conflicting results.^4^, ^7^, ^8^ Saliva is an interesting source of α‐syn because Lewy pathology has been noted in salivary glands,^9^ but studies of salivary α‐syn have produced contradictory results, with levels either increased or not different between PD and HC.^8^, ^10^ There are no studies of which we are aware that investigate the relationships among CSF, plasma, and salivary α‐syn in PD.

Several studies have examined CSF markers of neurodegeneration in PD (eg, α‐syn, beta‐amyloid [Aβ] 1‐40 and 1‐42, total tau [t‐tau], and phosphorylated tau [p‐tau] concentrations), but most studies focus on either de novo PD patients (eg, deprenyl and tocopherol antioxidative therapy of parkinsonism [DATATOP] or Parkinson's Progression Markers Initiative [PPMI]), or specifically on cognitive measures in PD cohorts.^1^, ^11^, ^12^, ^13^, ^14^, ^15^, ^16^ Of this work, the prevailing evidence is that CSF α‐syn is reduced in PD and that subspecies of α‐syn oligomers may distinguish PD from controls. Furthermore, Aβ and tau may provide useful insight into prognosticating cognitive decline in PD. To date, few studies have examined CSF α‐syn, Aβ, t‐tau, and p‐tau obtained simultaneously in moderate‐advanced “typical” PD cohorts or investigated how they relate to specific motor and nonmotor features of PD, such as motor subtype, sleep, and other nonmotor features.

BioFIND, a cross‐sectional, observational study of moderate‐advanced PD patients and matched HCs evaluated with standardized clinical and biospecimen acquisition protocols, provides a unique resource for examining biomarkers in a well‐characterized, “typical” (including good response to levodopa) PD population.^17^ The aim of our study was 3‐fold: (1) examine relationships among (a) CSF markers of neurodegeneration (ie, α‐syn, Aβ_1‐42_, t‐tau, p‐tau) in PD and HC in the BioFIND cohort and (b) in PD participants, the relationships among CSF, plasma, and saliva α‐syn levels; (2) determine differences between PD and HC in CSF α‐syn, Aβ_1‐42_, tau, and p‐tau and in plasma and saliva levels of α‐syn; and (3) investigate the associations of CSF markers with specific motor and nonmotor features of PD.

## Methods

### BioFIND Study and Design

BioFIND includes moderate‐advanced PD participants and HCs enrolled at 8 sites in the United States.^17^ PD patients had “typical” features, meeting United Kingdom PD Society Brain Bank clinical diagnostic criteria and having all 3 classic parkinsonian motor signs (ie, bradykinesia, rigidity, resting tremor) by history or examination; represented all Hoehn and Yahr stages; had disease durations ≥ 4 years and onset between ages 50 to 75 years; and demonstrated well‐established responses to dopaminergic agents and/or amantadine. Patients were excluded if they had features of atypical or secondary parkinsonian syndromes; a history of DBS or ablative brain surgery; a history of cancer (except basal or squamous cell skin cancers) within 5 years preceding enrollment; autoimmune, liver, or hematological disorders; or conditions precluding lumbar puncture. HCs were group matched by age and sex to PD patients, were free of any known neurological disorders, and scored ≥ 26 on the Montreal Cognitive Assessment (MoCA).^18^ Other exclusion criteria for HCs were similar to those for PD patients. Controls were excluded if they had a first‐degree family member with PD. Additional details regarding the study are discussed in the BioFIND paper.^17^

### Evaluations

Clinical data and biospecimen collection occurred on 2 visits (baseline, [V1] and follow‐up within 2 weeks of baseline, [V2]). For PD patients, V1 was performed in the on state (1‐3 hours after the last PD medication dose) and V2 was performed in the practically defined off state (early morning before PD medications and approximately 12 hours after the last dose the night before). V1 included collection of blood for DNA and plasma and assessments including the International Parkinson and Movement Disorder Society–Sponsored revision of the UPDRS (MDS‐UPDRS) parts I (nonmotor experiences of daily living), II (motor experiences of daily living), III (motor examination), and IV (motor complications) for PD patients and part III only for HCs.^19^ V2 included collection of blood for RNA and plasma, CSF, saliva (added after study startup), and MDS‐UPDRS part III in PD patients. All PD and HCs either fasted or had a low‐fat diet on the morning of V2. Assessments included demographics, family history of PD, medical/neurological histories, medications, neurological exams, and the MDS‐UPDRS,^19^ MoCA,^18^ and Rapid Eye Movement Behavior Sleep Disorder (RBD) Questionnaire^20^ and for PD patients, the Modified Schwab and England Activities of Daily Living Scale.^21^

### Motor and Nonmotor Features

For PD patients, motor phenotype was classified as tremor dominant (TD), postural instability/gait difficulty (PIGD), and indeterminate,^22^, ^23^ calculated from MDS‐UPDRS part III motor scores (V2, off) plus pertinent history questions from MDS‐UPDRS part II (2.10 tremor, 2.12 walking and balance, 2.13 freezing; V1 and acquired only once during the study).^23^ Other motor and nonmotor features were examined using the published factor structure of the MDS‐UDPRS for parts I to IV.^19^ Cognitive function was measured by MoCA total and subdomain scores (eg, visuospatial/executive, naming, attention, language, abstraction, delayed recall, orientation). RBD was examined using the RBD questionnaire total score, with a cut‐off of > 5 as indicative of RBD symptoms.

The study was approved by the institutional review boards for the University of Rochester Clinical Trials Coordination Center and study sites. Written, informed consent was obtained from the study participants.

### Biospecimen Analyses

Concentrations of α‐syn in plasma, CSF, and saliva samples were analyzed using ELISA assays (BioLegend, cat. 844101). The concentration of α‐syn in each sample was determined by interpolation of values against the standard curve established by the reference standards (range 1500 pg/ml‐6.1 pg/ml) using a 4‐parameter regression. Recombinant α‐syn from rPeptide was used as the standard in this assay. Each sample was analyzed in duplicate at appropriate dilutions (1/20 for CSF, 1/200 for plasma, 1/4 for saliva). Concentrations of hemoglobin in CSF, plasma, and saliva samples were analyzed using ELISA assays (Bethyl Laboratories, cat. E80‐134). Reference standards used in the assay ranged from 7.5 ng/ml to 125 ng/ml. The concentration of hemoglobin in each sample was determined by interpolation of values against the standard curve established by the reference standards using a 4‐parameter regression. The assay has been validated in multiple matrices including in CSF, plasma, and saliva (BioLegend manual November 11, 2016). Interassay precision and spike/recovery data for saliva are depicted (Supplementary Figure).

Simultaneous analysis of Aβ_1‐42_, t‐tau, and p‐tau in CSF was performed using a highly standardized micro‐bead based research‐use‐only immunoassay (INNO‐BIA Alz Bio3 kits; Fujirebio, Ghent, Blegium).^11^ CSF samples without dilution were measured in 6 analytical runs in 96‐well format, with each set of analyte calibration standards and quality control samples. A biomarker result was defined as the average of the duplicate concentration values. If a biomarker result did not meet specified criteria for acceptance, the result was not included in the final dataset. Two Aβ_1‐42_ and 6 t‐tau results had microbead counts <50 and 1 p‐tau_181_ result had duplicate precision (%CV) > 25%; therefore, these 9 results were invalidated.

### Statistical Analysis

The analyses were conducted in IBM SPSS Statistics version 23 (IBM Corp., Armonk, New York). Demographic data, disease characteristics, and biomarker concentrations were compared between PD and HC groups using *t* tests. Gender and dichotomous variables (eg, RBD scores) were compared between groups using chi‐square tests. Between‐subjects tests were used to examine MoCA total and subdomain scores controlling for age and gender and CSF biomarker levels in motor phenotypes controlling for age, gender, and duration of disease. Pearson and Spearman rho correlations were used to examine the relationships among biofluids, CSF biomarkers, and for CSF with motor and nonmotor variables, as appropriate and controlling for age, gender, and education. Regression models controlling for age, gender, and education were used to examine CSF biomarker predictors of PD diagnosis.

## Results

### Participant Characteristics

Clinical characteristics of BioFIND participants who provided at least one of the biomarker specimens are presented in Table 1. Gender and education years did not differ significantly between PD and HC groups. PD patients were older than HCs (*P* < .01), with mean age of disease onset 59.9 years, disease duration 8.34 years, MDS‐UPDRS part III total motor score (off) 39.13, and a median Hoehn and Yahr stage of 2. PD motor phenotypes included 51.3% TD, 36.5% PIGD, and 12.2% indeterminate. MoCA total scores were significantly lower in PD patients when compared with HCs (*P* < .001) as expected because HCs with MoCA scores ≤ 26 were excluded. Of the PD participants, 45.2% had RBD scores > 5, compared to 6.8% of HCs (*P* < .001).

**Table 1:** Demographic and clinical features of the BioFIND study cohort

	Healthy controls, n = 88	PD, n = 115	*P* value
Age, y	65.64 (7.36)	68.24 (6.40)	<.01
Gender, % male	51.10	62.60	.10
Age at PD onset, y		59.90 (6.17)	
Duration of PD, y (SD)		8.34 (3.09)	
Education, y	17.00 (3.06)	16.98 (2.97)	.97
LEDD, mg/day		740.50 (385.76)	
MDS‐UPDRS part I		9.47 (5.61)	
MDS‐UPDRS part II		11.10 (6.29)	
MDS‐UPDRS part III (off)	1.58	39.13 (13.19)	<.001
MDS‐UPDRS part IV		3.50 (2.89)	
Hoehn and Yahr stage		2.18 (0.67)	
RBD score	2.42 (1.89)	5.18 (3.39)	<.001
RBD > 5, %	6.80	45.20	<.001
TD, %		51.30	
PIGD, %		36.50	
Indeterminate, %		12.20	
MoCA total score	27.81 (1.43)	26.76 (2.56)	<.001
MoCA visuospatial/executive	4.40 (0.72)	4.18 (0.94)	.07
MoCA naming	2.95 (0.21)	2.88 (0.33)	.06
MoCA attention	5.84 (0.43)	5.79 (0.52)	.22
MoCA language	3.60 (0.72)	3.36 (1.00)	.04
MoCA abstraction	1.89 (0.32)	1.87 (0.36)	.87
MoCA delayed recall	3.95 (1.09)	3.45 (1.29)	<.001
MoCA orientation	5.99 (0.11)	5.92 (0.27)	<.01
MDS‐UPDRS part I, factor 1		8.14 (4.49)	
MDS‐UPDRS part I, factor 2		1.33 (1.64)	
MDS‐UPDRS part II, factor 1		4.61 (2.98)	
MDS‐UPDRS part II, factor 2		1.82 (1.25)	
MDS‐UPDRS part II, factor 3		4.67 (3.22)	
MDS‐UPDRS part III, factor 1		9.71 (4.33)	
MDS‐UPDRS part III, factor 2		9.37 (4.12)	
MDS‐UPDRS part III, factor 3		7.10 (3.02)	
MDS‐UPDRS part III, factor 4		4.37 (2.24)	
MDS‐UPDRS part III, factor 5		5.19 (2.63)	
MDS‐UPDRS part III, factor 6		2.92 (2.28)	
MDS‐UPDRS part III, factor 7		5.83 (2.96)	
MDS‐UPDRS part IV, factor 1		2.91 (2.55)	
MDS‐UPDRS part IV, factor 2		0.58 (0.80)	

Data are presented as mean (standard deviation) unless otherwise noted. RBD, Rapid Eye Movement Behavior Sleep Disorder; TD, tremor dominant; PIGD, postural instability/gait difficulty.

### Comparisons of CSF, Plasma, and Saliva Biomarkers in the BioFIND Cohort

**Figure 1:**
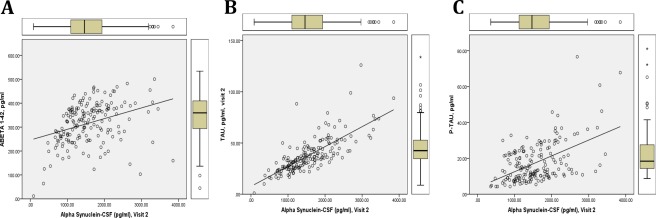
Correlations among CSF alpha‐synuclein (α‐syn), beta‐amyloid (Aβ) 1‐42, tau, and phosphorylated tau (p‐tau) in PD and healthy controls. Regression correlation among CSF α‐syn and Aβ1‐42, tau, and p‐tau in BioFIND cohort (n = 88, healthy controls; n = 115, PD). CSF α‐syn concentrations significantly correlate with all CSF biomarkers: (**A**) Aβ1‐42 (*r* = 0.41), (**B**) tau (*r* = 0.81), (**C**) p‐tau (*r* = 0.43); all *P* values < .001. [Color figure can be viewed at wileyonlinelibrary.com]

In the complete BioFIND cohort (PD and HC), CSF α‐syn concentrations were most strongly correlated with t‐tau (*r* = 0.81) and modestly with p‐tau (*r* = 0.43) and Aβ_1‐42_ (*r* = 0.41; *P*s < .001; Fig. 1). Age was weakly, but significantly, correlated with CSF measures including hemoglobin (*r* = 0.18, *P* = .017), α‐syn (*r* = 0.15, *P* = .043), and t‐tau (*r* = 0.20, *P* < .01). Among PD participants only, correlations between CSF α‐syn concentrations and Aβ_1‐42_, t‐tau, and p‐tau remained consistent with those found in the complete BioFIND cohort.

Hemoglobin and α‐syn levels were modestly correlated in saliva (*r* = 0.41, *P* = .04), but not in plasma (*r* = −0.28, *P* = .16) or CSF (*r* = −0.12, *P* = .57) in the BioFIND cohort. Saliva samples with hemoglobin concentrations > 1200 ng/ml were excluded to avoid potential impact of contaminant hemoglobin on α‐syn levels.^11^, ^24^ We did not exclude participants based on CSF or plasma hemoglobin levels because there was no significant correlation between hemoglobin and α‐syn levels in plasma and CSF.

Comparing α‐syn across the different biofluids in the BioFIND cohort, we did not find significant correlations for α‐syn concentrations between CSF and plasma (*r* = −0.08, *P* = .70, n = 177), CSF and saliva (*r* = −0.13, *P* = .53, n = 41), or plasma and saliva (*r* = 0.15, *P* = .48, n = 48). Likewise, among PD patients, there were no significant correlations between α‐syn concentrations in CSF and plasma (*r* = −0.15, *P* = .14, n = 98), CSF and saliva (*r* = −0.41, *P* = .87, n = 20), or plasma and saliva (*r* = −0.25, *P* = .26, n = 22).

### Comparisons of Biomarkers Between PD and HC

**Figure 2:**
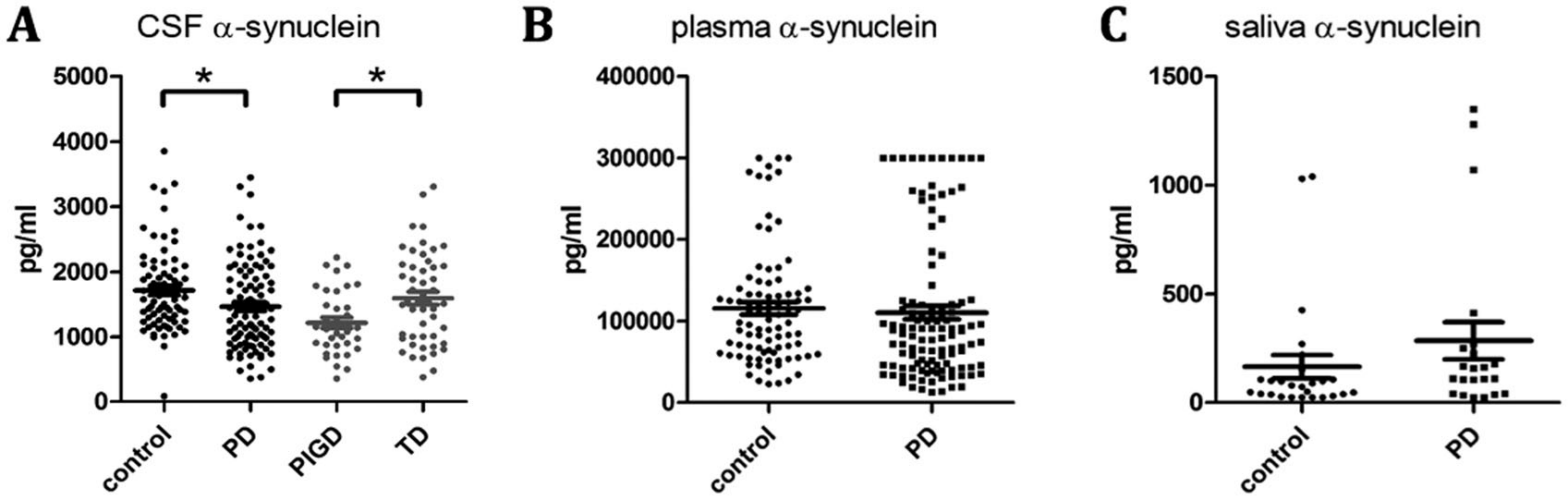
Comparisons of alpha‐synuclein in CSF, plasma, and saliva in PD and healthy controls. Measurements of alpha‐synuclein concentrations in (**A**) CSF between PD patients and healthy controls and between PD patients with postural instability/gait difficulty (PIGD) and tremor dominant (TD) motor phenotypes, (**B**) plasma between PD patients and healthy controls, and (**C**) saliva between PD patients and healthy controls (*P ≤ .01).

CSF α‐syn (*P* = .01) levels were lower in PD patients when compared with HCs (Table 2, Fig. 2). The α‐syn levels in plasma (*P* = .64) and saliva (*P* = .23), however, did not differ between PD and HCs. CSF Aβ_1‐42_ (*P* < .01) and p‐tau (*P* = .05) levels were lower in PD patients when compared with HCs, but t‐tau (*P* = .08) levels did not differ between PD and HCs (Table 2). In regression models adjusted for age, gender, and education years, the CSF biomarkers were significant predictors of PD diagnosis (Table 3).

**Table 2:** Comparison of CSF biomarkers in the BioFIND study cohort

Biomarkers	Healthy controls	PD	*P* value
CSF^a^			
α‐syn, pg/mL	1713.59 (637.16)	1466.25 (669.20)	.01
Aβ_1‐42_, pg/mL	334.75 (89.40)	296.31 (79.16)	<.01
t‐tau, pg/mL	42.06 (23.32)	37.15 (16.42)	.08
p‐tau_181_, pg/mL	20.61 (17.23)	16.42 (11.10)	.05
t‐tau/Aβ_1‐42_	0.15 (0.19)	0.13 (0.08)	.36
p‐tau_181_/Aβ_1‐42_	0.06 (0.06)	0.07 (0.12)	.28
Hb, ng/ml^a^	165.73 (290.50)	157.04 (296.66)	.85
Plasma^b^			
α‐syn, pg/mL	115,920.30 (71,392.10)	110,603.05 (87,761.66)	.64
Saliva^c^			
α‐syn, pg/mL	165.97 (272.25)	285.42 (400.13)	.23

Data are presented as mean (standard deviation) unless otherwise noted. α‐syn, alpha‐synuclein; Aβ, beta‐amyloid; t‐tau, total tau; p‐tau, phosphorylated tau.

a CSF sample size: healthy controls, n = 76‐79; PD, n = 98‐106 (range).

b Plasma sample size: healthy controls, n = 88; PD, n = 118.

c Saliva sample size: healthy controls, n = 26; PD, n = 22.

**Table 3:** Regression coefficients of CSF biomarkers in PD

	β‐coefficient	*t* value	*P* value	95% CI
α‐syn	290.39	2.85	.005	89.47‐491.30
Aβ_1‐42_	36.67	2.84	.005	11.21‐62.13
t‐tau	6.13	2.07	.04	0.29‐11.96
p‐tau	4.54	2.05	.04	0.16‐8.71

CI, confidence interval; α‐syn, alpha‐synuclein; Aβ, beta‐amyloid; t‐tau, total tau; p‐tau, phosphorylated tau.

### Relationship of CSF Biomarkers With Motor and Nonmotor Features in PD

None of the CSF biomarkers correlated with, or predicted, MDS‐UPDRS total motor scores. However, CSF α‐syn levels differed among motor phenotypes (*P* = .01), with lower concentrations in participants with PIGD phenotypes (1215.40 [491.56] pg/ml) than indeterminate (1682.99 [806.14] pg/ml) and TD phenotypes (1594.85 [702.45] pg/ml). Post hoc comparisons revealed differences between PIGD and TD (*P* < .01; Fig. 2) and PIGD and indeterminate phenotypes (*P* = .03). The significant difference between motor subtypes was noted when covariates of age, gender, and disease duration were considered. CSF Aβ_1‐42_, t‐tau, and p‐tau did not differ significantly among the PD motor phenotypes. None of the CSF biomarkers correlated with factors of the MDS‐UPDRS parts I to IV.

The only CSF biomarker associated with cognition was Aβ_1‐42_, which correlated weakly with MoCA remote recall (Spearman rho = 0.23, *P* = .02) and attention subscores (Spearman rho = −0.19, *P* = .05). There were no significant correlations between any of the CSF biomarkers and dichotomized RBD scores in the PD patients.

## Discussion

This cross‐sectional, observational study examined the levels of α‐syn across multiple biofluid matrices (ie, CSF, saliva, and plasma) and multiple CSF neurodegenerative biomarkers (ie, α‐syn, Aβ_1‐42_, t‐tau, and p‐tau) in a large sample of “typical,” moderate‐advanced PD and matched healthy control participants. CSF α‐syn strongly correlated with t‐tau and modestly, but significantly, with Aβ_1‐42_ and p‐tau. α‐syn levels in different biofluids such as CSF, plasma, and saliva did not significantly correlate with each other. Overall, CSF α‐syn, Aβ_1‐42_, and p‐tau concentrations were lower in PD when compared with healthy control participants, whereas t‐tau levels did not significantly differ between groups. Furthermore, the concentration of CSF α‐syn, but not the other CSF neurodegenerative markers, was significantly lower in those PD patients with PIGD motor phenotypes than in TD and indeterminate phenotypes. Finally, CSF Aβ_1‐42_ levels significantly correlated with aspects of cognition on the MoCA examination.

Our findings provide insights into the α‐syn levels across multiple biofluid matrices. Peripheral manifestation of Lewy type pathology has been explored as a diagnostic marker. One study noted a weak correlation (*r* = 0.197) of CSF and total plasma α‐syn,^4^ but we did not find a significant correlation of α‐syn levels in peripheral biofluids (plasma and saliva) with α‐syn levels in CSF in our cohort, perhaps because of the differences in biofluid methodologies or sample characteristics. Plasma and saliva α‐syn levels did not significantly differentiate PD from healthy control participants. Thus, CSF α‐syn has greater diagnostic utility for PD than peripheral α‐syn despite its more invasive nature for obtaining samples.

Consistent with published literature including a study of 63 de novo PD and 32 healthy control participants in PPMI, CSF α‐syn levels in PD strongly correlated with t‐tau, and to a lesser degree, p‐tau.^11^ In BioFIND, we also identified a modest correlation of CSF α‐syn and Aβ_1‐42_, which supports findings from a larger PPMI study of 660 participants (*r* = 0.35, *P* < .01).^25^ These findings suggest that CSF α‐syn, Aβ_1‐42_, and tau may interact on cellular and pathophysiological levels in PD. Interactions of α‐syn and tau proteins have been described in animal models and postmortem brain studies, including colocalization of tau‐positive tangles and α‐syn‐positive Lewy bodies,^26^, ^27^ and α‐syn and Aβ_1‐42_ may have a synergistic relationship.^28^, ^29^ A greater understanding of the molecular mechanisms of overlapping proteinopathies and their underlying pathology in PD, along with relevant biomarkers, will contribute to the development and validation of biomarkers used for diagnosis, prognosis, and therapeutic monitoring in PD.

In agreement with previous reports, CSF α‐syn levels were lower in PD than in HCs and, in classification and regression models, distinguished these groups from each other.^5^, ^30^, ^31^, ^32^ In the BioFIND cohort, CSF t‐tau levels did not differ significantly between groups, although p‐tau levels demonstrated borderline significance (*P* = .05). Aβ_1‐42_ also differentiated PD patients when compared with healthy controls. We recognize, however, that many studies represent healthy controls as the ceiling of health, such as our enrollment criteria of controls with MoCA scores ≥ 26; these groupings may introduce potential bias by detecting greater differences between disease and healthy states. However, it is interesting to note that CSF Aβ_1‐42_ may relate to certain cognitive domains such as remote memory; this finding supports CSF Aβ_1‐42_ as an important potential biomarker for cognitive impairment in PD.^12^, ^15^, ^16^, ^33^, ^34^, ^35^

It is notable that CSF α‐syn levels did not correlate with several disease‐related characteristics, including MDS‐UPDRS total motor scores (part III), motor complications (part IV), and motor experiences of daily living (part II). Furthermore, CSF α‐syn did not correlate with nonmotor features including RBD, cognition as measured by the MoCA, or nonmotor experiences of daily living (MDS‐UPDRS part I). The lack of a relationship between RBD and CSF α‐syn in our cohort differs from a study in which oligomeric α‐syn levels were elevated in the CSF and serum in PD patients with RBD when compared with PD patients without RBD and controls. The different methodologies used and the more advanced PD participants included in our study may contribute to different study findings.^36^ CSF α‐syn levels may be useful for distinguishing the disease state, but may not be strong biomarkers for disease progression once already at moderate‐advanced stages.

CSF α‐syn levels differed among the PD motor phenotypes, with lower levels in the PIGD subtype. Although lower CSF α‐syn levels may not predict motor scores, they may be useful in distinguishing motor phenotypes and for prognosticiation because PIGD deficits may portend a worsened motor and cognitive course of PD. The relationship of CSF α‐syn, but not other CSF markers, to PIGD motor phenotype in PD also occurs in early PD as described in the PPMI cohort.^25^ Although suggestive of a potential prognostic value, longitudinal studies are needed for confirmation.^11^

The strengths of our study include well‐characterized and “typical” PD patients, rigorously collected and detailed clinical and biospecimen data, and simultaneous measurement of multiple CSF neurodegenerative markers and of α‐syn across multiple biofluid sources. We probed relationships of CSF biomarkers with motor and nonmotor variables, such as RBD and MDS‐UPDRS factors, which have received limited attention to date. We note several study limitations, including its cross‐sectional design and lack of clinico‐pathological correlations, which preclude our ability to determine relationships between biomarkers and disease progression or neuropathology. RBD assessment was based on self‐report without confirmation by polysomnography, and cognition was measured with a screening questionnaire rather than a full cognitive battery. Despite this, our findings provide direction for design of more in‐depth future studies. Lastly, although our CSF and plasma sample sizes are large, the number of salivary specimens was small because this assessment was added after the study had begun, and thus larger studies are needed.

The BioFIND cohort offers a unique opportunity to compare α‐syn across different biofluids. In our cohort, α‐syn levels in different biofluids such as CSF, plasma, and saliva did not significantly correlate with each other. In addition, the finding that CSF α‐syn levels are associated with PIGD motor phenotype suggests that this marker may also be useful for providing prognostic information for PD patients.

## Author Roles

1) Research project: A. Conception, B. Organization, C. Execution; 2) Statistical Analysis: A. Design, B. Execution, C. Review and Critique; 3) Manuscript: A. Writing of the first draft, B. Review and Critique.

J.G.G.: 1A, 1B, 1C, 2A, 2B, 2C, 3A, 3B

H.A.: 2A, 2B, 2C

A.A.: 1A, 1B, 1C, 3A, 3B

A.N.: 1A, 1B, 1C, 2A, 2B, 2C, 3B

R.N.A.: 1A, 1B, 1C, 2A, 2B, 2C, 3A, 3B

L.M.S.: 1A, 1B, 1C, 3B

P.T.: 1A, 1B, 1C, 3B

T.X.: 1A, 1B, 1C, 3B

P.T.: 1A, 1B, 1C, 3B

C.H.: 1A, 1B, 1C, 3B

P.H.: 1A, 1B, 1C, 3B

S.F.: 1A, 1B, 1C, 3B

M.H.S.H.: 1A, 1B, 1C, 3B

M.F.: 1A, 1B, 1C

V.A.: 1A, 1B, 1C

A.N.R.: 1A, 1B, 1C

M.S.: 1A, 1B, 1C

C.S.F.: 1A, 1B, 1C

K.G.: 1A, 1B, 1C

U.J.K.: 1A, 1B, 1C, 2A, 2B, 2C, 3B

## Financial disclosures of all authors (for the preceding 12 months)

J.G.G. reports the following: consulting and advisory board membership with honoraria: Acadia, Aptinyx, Biogen; grants and research: Acadia, Biotie (site‐PI), Consolidated Anti‐Aging, National Institutes of Health, National Parkinson Foundation, The Michael J. Fox Foundation, Rush University, Agency Honoraria: American Academy of Neurology, International Parkinson and Movement Disorder Society, MedEdicus; employment: Rush University. H.A. reports the following: consulting and advisory board membership with honoraria: The Michael J. Fox Foundation; Employment: Columbia University. A.A. reports the following: consulting and advisory board membership with honoraria: advisory board for Jazz Pharmaceuticals; grants and research: NIH NINDS, NIA, Parkinson's disease Foundation, Rehabilitation Research Resource to Enhance Clinical Trials, The Michael J. Fox Foundation, AbbVie, Jazz Pharmaceuticals, Axovant Sciences, Ltd.; Employment: University of Alabama at Birmingham. A.N. reports Employment: The Michael J. Fox Foundation. L.M.S. reports funding from the Michael J. Fox Foundation to complete the analysis of CSF Aβ_1‐42_, t‐tau and p‐tau in this study; consulting and advisory board membership with honoraria: Eli Lilly, Roche; grants and research: The Michael J. Fox Foundation, NIH(NIA), provide QC oversight for AD CSF immunoassays in the past for AlzBio3 immunoassay (Fujirebio) and currently for the Roche Elecsys fully automated CSF AD immunoassays; Employment: University of Pennsylvania. P.T. is an employee of BioLegend, the entity responsible for the manufacture and commercialization of the alpha‐synuclein ELISA (Cat. 844101) used in this study. BioLegend received funding from the Michael J. Fox Foundation to complete the analysis of alpha‐synuclein and hemoglobin in samples collected from the study cohort. P.T. reports the following: grants and research: BioLegend received funding from the Michael J. Fox Foundation to complete the analysis of alpha‐synuclein and hemoglobin in samples collected from the study cohort; employment: BioLegend. R.N.A. reports the following: grants and research: Parkinson's Disease Foundation, the National Institutes of Health (K02NS080915), and The Michael J Fox Foundation; agency honoraria: Genzyme/Sanofi, Denali, and Prophase; employment: Columbia University. T.X. reports the following: grants and research: The Michael J Fox Foundation, Bristol‐Myers Squibb; agency honoraria: CVS/Caremark; employment: University of Chicago Medicine. P.T. reports the following: grants and research: NIH, Bristol‐Myers Squibb, Biogen, Northwestern University, The Michael J. Fox Foundation, Biotie, Kyowa, and University of Minnesota; employment: University of Minnesota and University of Minnesota Physicians. C.H. the following: consulting and advisory board membership with honoraria: ACADIA ‐ ad hoc advisory board participation; US WorldMeds: ad hoc advisory board participation; Gerson Lehman Group: consultation; grants and research: NIH/NINDS; NIH/NOIT; New York State Department of Health /Empire State Stem Cell Board; employment: Weill Cornell Medical College. P.H. reports the following: grants and research: The Michael J. Fox Foundation; employment: Oregon Health Science University. S.F. reports the following: grants and research: Teva Pharmaceuticals; employment: Beth Israel Deaconess Medical Center. M.H.S.H. reports the following: consulting and advisory board membership with honoraria: Delsys Inc, Acorda; grants and research: MJFF, NIH, Acorda, Auspex; employment: Boston University. M.F., V.A., and A.N.R. report the following: employment: The Michael J. Fox Foundation. M.S., C.S.F., and K.G. report the following: employment: National Institute of Neurological Disorders and Stroke. U.J.K. reports the following: consulting and advisory board membership with honoraria: PF SAB, APDA SAB; grants and research: NIH R01 NS101982, R03 NS096494, U01 NS100600, DoD/CDMRP Discovery Award (PR161817), The Michael J. Fox Foundation, and Parkinson's Foundation; agency honoraria: NIH study section, PF, The Michael J. Fox Foundation grant review; employment: Columbia University.

## Supporting information

Additional Supporting Information may be found in the online version of this article at the publisher's website

Supporting Information 1
