# Supplementary figures and images for: Cerebrospinal fluid, plasma, and saliva in the BioFIND study: Relationships among biomarkers and Parkinson's disease Features
Source: Mov Disord. 2017 Dec 4;33(2):282–8. doi: 10.1002/mds.27232 (PMC5836918; doi:10.1002/mds.27232)

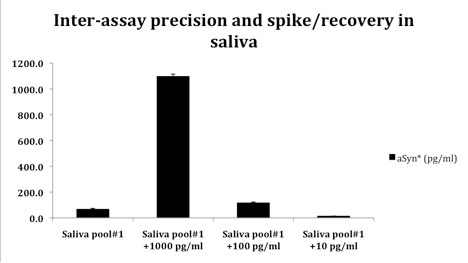

Supplement: Supplementary file 1 — Supporting Information 1 [file MDS-33-282-s001.tif]
